# The innate immune system and neurogenesis as modulating mechanisms of electroconvulsive therapy in pre-clinical studies

**DOI:** 10.1177/0269881120936538

**Published:** 2020-07-10

**Authors:** Juliette Giacobbe, Carmine M Pariante, Alessandra Borsini

**Affiliations:** Department of Psychological Medicine, King’s College London, London, UK

**Keywords:** Electroconvulsive therapy (ECT), neurogenesis, neuroinflammation, depression, glial activation

## Abstract

**Background:**

Electroconvulsive therapy (ECT) is a powerful and fast-acting anti-depressant strategy, often used in treatment-resistant patients. In turn, patients with treatment-resistant depression often present an increased inflammatory response. The impact of ECT on several pathophysiological mechanisms of depression has been investigated, with a focus which has largely been on cellular and synaptic plasticity. Although changes in the immune system are known to influence neurogenesis, these processes have principally been explored independently from each other in the context of ECT.

**Objective:**

The aim of this review was to compare the time-dependent consequences of acute and chronic ECT on concomitant innate immune system and neurogenesis-related outcomes measured in the central nervous system in pre-clinical studies.

**Results:**

During the few hours following acute electroconvulsive shock (ECS), the expression of the astrocytic reactivity marker glial fibrillary acidic protein (GFAP) and inflammatory genes, such as cyclooxygenase-2 (COX2), were significantly increased together with the neurogenic brain-derived neurotrophic factor (BDNF) and cell proliferation. Similarly, chronic ECS caused an initial upregulation of the same astrocytic marker, immune genes, and neurogenic factors. Interestingly, over time, inflammation appeared to be dampened, while glial activation and neurogenesis were maintained, after either acute or chronic ECS.

**Conclusion:**

Regardless of treatment duration ECS would seemingly trigger a rapid increase in inflammatory molecules, dampened over time, as well as a long-lasting activation of astrocytes and production of growth and neurotrophic factors, leading to cell proliferation. This suggests that both innate immune system response and neurogenesis might contribute to the efficacy of ECT.

## Introduction

Electroconvulsive therapy (ECT) is a powerful and fast-acting treatment intervention, used particularly in treatment-resistant patients with severe depression. ECT has been shown to be more effective on depressive symptoms than classic pharmacological treatment in unipolar (The UK ECT Review Group, 2003) and bipolar depression (Schoeyen et al., 2015), and is generally associated with lower rates of relapse in combination with pharmacotherapy in comparison with pharmacological intervention alone (Elias et al., 2017; Jelovac et al., 2013). While concerns have been raised with respect to side-effects on cognitive functions, such as memory, these seem to be transient (Kirov et al., 2016; Vasavada et al., 2017) and possibly related to the severity of the disorder (Andrade et al., 2016). Overall, ECT is considered a safe therapeutic option for patients who do not respond to antidepressant medications and thus are at risk of experiencing prolonged periods of severe depression, leading to impaired quality of live and even life-threatening consequences, including suicidal acts and physical deterioration from lack of food and water intake. In general, there is evidence of increased quality of life in elderly patients suffering from depression (McCall et al., 2018). However, in contrast with the consensus on its clinical benefits, the mechanisms underlying the rapid effects of ECT remain to be elucidated.

ECT is widely thought to act on neuroplasticity and neurogenesis-related processes, which are part of the pathophysiological processes leading to depression (Innes et al., 2018). More specifically, depression is typically associated with alterations related to the hippocampus, a neurogenic region critical to memory formation and emotion processing. In patients, reductions in hippocampal volume can be observed (Bremner et al., 2000), and in animal models, decreased proliferation and differentiation of new-born neurons have been reported in the hippocampal neurogenic niche (David et al., 2009; Malberg and Duman, 2003), contributing to cognitive changes observed in animal studies of depression. ECT has been able to reverse reduced hippocampal volume in patients (Tendolkar et al., 2013) and animal models, where electroconvulsive shock (ECS) is used to model the human intervention (Perera et al., 2007). In terms of synaptic plasticity, ECT is thought to increase the excitability of limbic structures critical to depression, including the hippocampus, where immature cells might be particularly sensitive (Rotheneichner et al., 2014). In patients, ECT increases levels of blood brain-derived neurotrophic factor (BDNF) (Brunoni et al., 2014), a factor relevant to cellular plasticity which triggers signalling cascades regulating the transcription of neuronal survival and growth factors (Björkholm and Monteggia, 2016; Zhang et al., 2014).

Impaired neurogenesis-related processes, in turn, might be influenced by other factors, such as inflammation. Higher expression of pro-inflammatory cytokines, including interleukin (IL)-1β, IL-6, or tumour necrosis alpha (TNF-α), causes reductions in hippocampal proliferation in vitro (Borsini et al., 2017) and in vivo, which has been reported alongside depressive-like behaviour (Tang et al., 2016). Changes in the activation of microglia, the immune cells of the central nervous system (CNS), have also been shown to be IL-1β-dependent and can contribute to neurogenesis impairments and to the manifestation of depressive-like symptoms (Goshen et al., 2008; Kreisel et al., 2013). In addition, increases in those cytokine levels are observed in the periphery in patients, particularly those who are treatment resistant (Salam et al., 2018; Strawbridge et al., 2015) and thus are the type of patients for whom ECT is especially indicated (Weiner and Reti, 2017).

Interestingly, there is some evidence that ECT influences immune function in patients, although the direction of the effect is not what could be expected. Specifically, even if the aforementioned pro-inflammatory markers are elevated in patients, ECT has been reported to increase serum cytokine expression (Lehtimaki et al., 2008) and to prime peripheral monocytes and enhance their production in response to inflammatory stimuli (Fluitman et al., 2011). TNF-α seems to only decrease peripherally in the long term, after several courses of ECT (Hestad et al., 2003). In animals, the effects of ECS on central inflammation have not fully been disentangled. For example, ECS increases astrocytic and microglial activation in some studies (Jansson et al., 2009) but also reduces this in others (Jinno and Kosaka, 2008). Cytokines levels are elevated in the hippocampus after ECS (Zhu et al., 2015), but these changes are time-dependent and not yet investigated in patients. Central inflammation in depression, if present, would be able to rapidly affect neurogenesis, as this process only takes place in distinct neurogenic niches in the CNS, such as the subgranular zone (SGZ) of the dentate gyrus in the hippocampus or the subventricular zone (SVZ) of the lateral ventricles (Borsini et al., 2015).

Despite the fact that changes in the immune system are known to influence neurogenesis, animal studies in the context of ECT (Guloksuz et al., 2014; van Buel et al., 2015) have for, the most part, only explored these two biological systems independently from each other. Therefore, the aim of this review is to compare the time-dependent consequences of acute and chronic ECT on concomitant immune system and neurogenesis-related outcomes measured in the CNS in pre-clinical studies. In line with previous literature in the field of neuroimmunology (Heneka et al., 2014), our search covered changes related to innate immune activation, encompassing both glial activation and inflammatory reactions. In order to shed light on these processes, we conducted review of available papers on these topics in PubMed and Google Scholar, and only identified a total of 15 pre-clinical studies assessing the effects of acute or chronic ECS on both innate immune system and neurogenesis-related outcomes.

## Results and discussion

### Innate immune system and neurogenesis outcomes assessed hours after acute or chronic ECS

In this section, the effects of acute or chronic ECS on innate immune system and neurogenesis outcomes will be discussed in the hours following the end of treatment. For the purpose of this review, acute ECS was defined as ECS applied for a duration of 3 days or less, and chronic ECS as ECS applied for 4 days or more. On average, chronic ECS courses lasted 10 days in the selected papers. Four studies investigated the actions of the treatment on glial activation (Table 1), six studies focused on changes in inflammatory molecules along with neurogenesis (Table 2), while only one study investigated both. Out of those 11 studies, three ran experiments on acute and chronic ECS and will appear in both sections.

**Table 1.** Astroglial activation and neurogenesis after acute and chronic ECS.

Article	Animals	Experimental manipulations	ECS frequency	Sacrifice/samples	Innate immune system finding	Neurogenesis finding	Behavioural finding
**Astroglial activation and neurogenesis**							
Conti et al. (2007) *	Sprague-Dawley rats	ECS, sleep deprivation or fluoxetine	4/day for 2 days	1h after last ECS	↗GFAP gene expression in PFC ↗AQP4 and glia maturation factor gene expression in locus coeruleus	↗BDNF gene expression in PFC, amygdala, HIPP ↗Homer1 gene expression in PFC, amygdala, HIPP, HYP	–
Okada-Tsuchioka et al. (2014)	Sprague-Dawley rats	ECS	1x	1h, 2h, 4h, 8h, 16h, 24h after ECS	↗GFAP mRNA in HIPP Peaks 16h after acute ECS	↗TSP-1 mRNA (involved in synaptogenesis) in HIPP Peaks 2h after acute ECS	–
			1/day for 10 days	1h, 2h, 24h after ECS	↗GFAP mRNA in HIPP Peaks 24h after chronic ECS at comparable levels to acute ECS	↗TSP-1 mRNA (involved in synaptogenesis) in HIPP Peaks 2h after chronic ECS	
Kragh et al. (1993)	Rats (N/A strand)	ECS with or with/ lidocaine injections 30 min after	12-30x (5/week for 2-6 weeks)	± 1h30-2h after last ECS	↗ GFAP concentration in HIPP, amygdala, piriform cortex (no lidocaine effect)	ECS ↘D3-protein (mature synapses) and trend ↘ NCAM (new synapses)	–
Madsen et al. (2005)	Sprague-Dawley rats	ECS	1/day for 10 days	BrdU 6h after the last 3 or final ECS, then sacrifice 2h later	No GFAP/BrdU+ cells in mPFC	No NeuN/BrdU+ cells in mPFC	–
Dwork et al. (2004)	Rhesus macaque	ECT	4/week for 6 weeks	3 days after last ECT	↗GFAP in frontal gyrus, HIPP, amygdala (qualitative due to sample size)	No MAP2 differences in any region	–
O’Donovan et al. (2014)	Sprague-Dawley rats	ECS Subcutaneous corticosterone injection	1/day for 10 days	11 days after last ECS	↗GFAP protein and mRNA in HIPP	↗BDNF expression in HIPP	↘ immobility in FST
Steward (1994)	C57BL/6 mice	ECS after EC lesion	1/day for up until 14 days	2, 4, 6, 8, 10, 12, 14 days after lesion 3 weeks after lesion	↗GFAP mRNA in DG (single ECS, then peaks up until 4 days post ECS)	ECS ↘Ach sprouting in the DG area receiving projections from the EC after lesion (days after + weeks after lesion)	–
Yao et al. (2015)	Sprague-Dawley rats	Chronic ECS	1/day for 6 days	BrdU 3 days prior, on day 1 and after ECS1 Sacrifice 5 weeks later (estimated)	↗GFAP (nm^3^)	↗BrdU, Neuro-D, DCX+ cells in DG	ECS ↘ short-term memory ↗long-term memory

↗ increase; ↘ decrease; * study appearing twice across tables; - not assessed.

Ach: cholinergic; AQP4: aquaporin 4; BDNF: brain-derived neurotrophic factor; BrdU: bromodeoxyuridine; DCX: doublecortin; DG: dentate gyrus; EC: entorhinal cortex; ECS: electroconvulsive shock; FST: forced swim test; GFAP: glial fibrillary acidic protein; HIPP: hippocampus; MAP2: microtubule-associated protein 2; mPFC: medial prefrontal cortex; NCAM: Neural Cell Adhesion Molecule; PFC: prefrontal cortex; TSP-1: thrombospondin-1.

**Table 2.** Inflammatory molecules and neurogenesis after acute and chronic ECS.

Article	Animals	Experimental manipulations	ECS frequency	Sacrifice/samples	Innate immune system finding	Neurogenesis finding	Behavioural finding
**Inflammatory molecules and neurogenesis**							
Amini et al. (2018)	Sprague-Dawley rats	ECS Single i.c.v. LPS (1x low dose, 4 days pre-ECS) or chronic LPS (4x ultra-low dose, every 4 days pre-ECS)	3x, at 3h intervals	1h after last ECS	ECS ↗NF-κB gene exp in HIPP ↗TLR4, SHIP1, TOLLIP, IRF3 mRNA and protein expression in HIPP of single and chronic LPS+ECS vs vehicle+ECS ECS ↗ NF-κB mRNA and protein, ↘ NF-κB in single LPS (mRNA and protein) and chronic LPS (mRNA, not protein) vs. vehicle+ECS ↗IFN-B mRNA in single and chronic LPS+ECS vs. vehicle+ECS ↗IL-10 mRNA in chronic LPS+ECS vs. vehicle+ECS ↘TNF-α mRNA in single and chronic LPS+ECS vs. vehicle+ECS No changes in IL-1β	ECS ↘ nissl-stained cells in CA1 ↗ nissl-stained cells in single and chronic LPS+ECS vs vehicle+ECS group in CA1 and DG In CA3, only with chronic LPS	↘ tonic-clonic seizures developed in csq to ECS in single and chronic LPS groups
Conti et al. (2007) *	Sprague-Dawley rats	ECS, sleep deprivation or fluoxetine	4/day for 2 days	1h after last ECS	↘CYP450 gene expression in HYP ↗COX2 gene expression in HYP ↘CXCL12 gene expression in HIPP ↘IL-16 gene expression in locus coeruleus	↗BDNF gene expression in PFC, amygdala, HIPP ↗Homer1 gene expression in PFC, amygdala, HIPP, HYP	–
Altar et al. (2004)	Sprague-Dawley rats	ECS	1x	4h after last ECS	↗c-Jun gene expression in FC ↗CYP450 gene expression in FC ↗IL-6 receptor gene expression in FC		–
			1/day for 10 days		↗COX2 gene expression in HIPP and FC	↗BDNF gene expression in HIPP and FC + BDNF-related pathways	
Imoto et al. (2017)	C57BL/6 mice	ECS	1x	6h or 24h after last ECS	↘ cytokine response genes after 6h (single and chronic)	↘ Calb1 and Tdo2 (mature GC markers) gene expression in DG after 6h Returned to normal 24h after	–
			4/week for up to 3 weeks		↘ IL-1 receptor 1 gene expression in DG (chronic)	↘ Calb1 and Tdo2 (mature GC markers) gene expression in DG after 6h Maintained for 14 days after chronic ECS ↗Calretinin expression (immature GC) at 24h ↗somatic excitability, ↘resting potential, ↘ EPSP at MF-CA3 synapses juvenile-like phenotype 24h after chronic ECS	
		Chronic ECS vs. chronic SSRI	4/week for up to 3 weeks	24h after last ECS	↗genes related to cytokine response (chronic), ↘IL-1 mediated signalling pathways in DG by both ECS and SSRI	↗nervous system, axon development, differentiation related genes in DG by both ECS and SSRI	
Chang et al. (2018)	CF-1 mice	ECS WT or Narp KO mice	1/day for 5 days	BrdU every 12h from day 3 of ECS Sacrificed 24h after ECS		ECS ↗c-fos in BLA, DG, NAcc, VMH, and mPFC, no ≠ between WT and KO ECS ↗BrdU+ cells in HIPP, no ≠ between WT and KO ECS ↗BDNF mRNA, protein expression in HIPP, no ≠ between WT and KO ECS did not influence DCX+ cells ECS ↗ DCX+ arborisations in WT but not KO	ECS ↘ TST and FST immobility in WT, not KO ECS ↗locomotor activity, no ≠ between WT and KO
		Chronic stress mice, WT or Narp KO (supplementary)					ECS ↘TST immobility in stressed WT but not stressed KO

↗ increase; ↘ decrease; * study appearing twice across tables; - not assessed.

BDNF: brain-derived neurotrophic factor; BLA: basolateral amygdala; BrdU: bromodeoxyuridine; Calb1: calbindin 1; COX2: cyclooxygenase 2; CXCL12: CXC motif chemokine ligand 12; CYP450: cytochrome P450; DCX: doublecortin; DG: dentate gyrus; ECS: electroconvulsive shock; EPSP: excitatory postsynaptic potential; FC: frontal cortex; FST: forced swim test; GC: granule cell; HIPP: hippocampus; i.c.v.: intracerebroventricular; IFN-B: interferon-beta; IL-10: interleukin 10; IL-16: interleukin 16; IL-1β: interleukin 1 beta; IL-6: interleukin 6; IRF3: interferon regulatory factor 3; KO: knockout; LPS: lipopolysaccharide; MAP2: microtubule-associated protein 2; mPFC: medial prefrontal cortex; NAcc: nucleus accumbens; NF-κB: nuclear factor-kappa B; PFC: prefrontal cortex; SHIP1: Src homology 2-containing inositol phosphatase-1; SSRI: selective serotonin reuptake inhibitor; Tdo2: tryptophan 2,3-dioxygenase; TLR4: toll-like receptor 4; TOLLIP: toll interacting protein; TST: tail suspension test; WT: wild type.

### Astroglial activation and neurogenesis

#### Acute ECS treatment

In both studies reporting measures of astroglial activation in response to acute ECS, treatment appeared to stimulate astrocyte activity as well as the expression of neurogenesis-related proteins. In the first study (Conti et al., 2007), administrating ECS four times a day for 2 days caused an increase in transcripts of glial fibrillary acidic protein (GFAP), a marker of astrocyte activation, in the prefrontal cortex of healthy rats an hour after the last treatment. This was accompanied by increased BDNF and Homer1 transcripts in the prefrontal cortex, as well as in the hippocampus and amygdala (Conti et al., 2007). Generally, BDNF is considered to indicate an activity of neurogenic processes, particularly through its main receptor tropomyosin receptor kinase B (TrkB) (Björkholm and Monteggia, 2016). Homer1 is a scaffolding protein highly expressed at the postsynaptic density which is rapidly upregulated after neuronal activation (Luo et al., 2012). Together, these changes indicate that astroglial activation and promotion of neurogenesis are rapidly activated by acute ECS.

Further suggesting a stimulating effect of ECS on astrocytes, the same study found other genes to be upregulated in the locus coeruleus, including aquaporin 4 (AQP4), a water channel highly expressed in astrocytes, and glia maturation factor (Conti et al., 2007). Interestingly, the functional effects of changes in AQP4 expression are difficult to interpret: in one study, downregulating AQP4 reduced IL-1β and TNF-α and prevented neurological deficit following hypoxia ischemia (Liu et al., 2017), thus suggesting a protective effect. However, others have reported detrimental effects following deletion of AQP4, reducing BDNF levels and antidepressant efficacy (Kong et al., 2009; Xu et al., 2015). Cytokines like IL-6 and interferon-alpha also downregulate AQP4 expression and adversely affect neurogenesis (Borsini et al., 2018), emphasising the pivotal role of astrocytes.

Similarly, in the second study (Okada-Tsuchioka et al., 2014), single administration of ECS elevated the mRNA expression of GFAP in healthy rat hippocampus from 8 to 24 h after the last treatment, when it started declining. Moreover, ECS increased mRNA levels of thrombospondin-1 (TSP-1) at earlier time points, with a peak 2 h after ECS (Okada-Tsuchioka et al., 2014). TSP-1 is a molecule secreted by astrocytes and playing a role in presynaptic maturation (Christopherson et al., 2005; Crawford et al., 2012). Therefore, these findings support the rapid glial and neurogenic stimulation occurring in response to acute ECS.

#### Chronic ECS treatment

When chronic ECS was applied, comparable results were described in terms of astrocytic activation, whereas various outcomes were reported to assess neurogenesis in three studies. In a previously mentioned study using rats undergoing ECS once a day for 10 days (Okada-Tsuchioka et al., 2014), the treatment upregulated GFAP mRNA expression in the hippocampus as soon as 1 h afterwards, and further increased it until 24 h afterwards. This was reported along with higher TSP-1 mRNA levels, peaking 2 h after the last ECS (Okada-Tsuchioka et al., 2014). In the second study (Kragh et al., 1993), it appeared that when ECS was applied five times a week for 2 to 6 weeks, the concentration of GFAP protein did increase in the hippocampus, amygdala, and piriform cortex of rats about 2 h after the last session. D3-protein, a marker of mature synapses, decreased in the piriform cortex, while the new synapse marker neural cell adhesion molecule (NCAM) did not (Kragh et al., 1993). Another study (Madsen et al., 2005), using ECS once a day for 10 days, did not find either newly proliferating cells co-labelled with the marker GFAP, as marker of stem cells sub-populations, or NeuN, a marker of mature neurons, in rat medial prefrontal cortex 8 h afterwards (Madsen et al., 2005). The fact that these findings are not consistent may be because they were in the piriform and prefrontal cortices, while neurogenesis is a process principally located in the neurogenic niches, even though immune changes in the brain can be more widespread. Of note, ECS could also cause changes in neurogenesis-related molecules, but not directly affect neuronal maturation and synaptic formation, putatively explaining why no differences were found in those studies.

Nonetheless, these chronic ECS studies reveal a trend of elevated GFAP expression after treatment, as do acute ECS studies. Astrocytes are known to be dysregulated in depressed patients, namely with a marked atrophy characterised by lower GFAP expression (Cobb et al., 2016). Antidepressant treatments, such as fluoxetine, reverse this and restore astrocytic protrusion length in vivo after chronic stress (Zhao et al., 2018). Astrocytes projections and BDNF have also been upregulated in mice after exercise, a practice which is recommended to patients with depression and known to promote neurogenesis (Fahimi et al., 2017). Moreover, mice expressing risk gene variants in astrocytes have reduced proliferation of progenitors, which was linked with anxiety-like behaviour and impaired social behaviour (Terrillion et al., 2017), which would reinforce the hypothesis that acute astrocytic activation might promote neurogenesis and eventually improve clinical symptoms of depression.

### Inflammatory molecules and neurogenesis

#### Acute ECS treatment

Four of the selected studies reported a general activation of inflammatory molecules and pathways together with neurogenesis in response to acute ECS up to 4 h after treatment. In a study using three ECS courses in a day to model epilepsy, the protein and mRNA expression of nuclear factor-kappa B (NF-κB) was increased and cell viability was decreased in rat hippocampus 1 h after the last treatment (Amini et al., 2018). However, this was prevented in the hippocampus when a single or chronic dose of lipopolysaccharide (LPS) was injected intracerebroventricularly prior to ECS. LPS also increased protein and mRNA levels of toll-like receptor 4 (TLR4), Src homology 2-containing inositol phosphatase-1 (SHIP1), toll interacting protein (TOLLIP), and interferon regulatory factor 3 (IRF3) after ECS, compared with vehicle group, and additionally increased the transcription of interferon-beta (IFN-β) and decreased that of TNF-α. They were also one of the few studies to assess behavioural outcomes, and noticed that LPS reduced the number of tonic–clonic seizures developed in response to ECS treatment (Amini et al., 2018). Although TLR4 inactivation has previously been proposed as a target to reduce seizures (Maroso et al., 2010), a recent study showed that stimulating the toll-like receptor 3 (TLR3), typically also considered pro-inflammatory and ictogenic, could actually prevent the emergence of seizures via IRF3/IFN-β rather than NF-κB signalling (Kostoula et al., 2019). The present results may suggest that mimicking baseline levels of inflammation, such as those observed in depression, through LPS activation of TLR4, could trigger immune pathways leading to neuroprotective effects and thus prevent the ECS-induced reduction in cells’ viability.

Two other studies, one of which was mentioned in the previous section (Conti et al., 2007), also assessed inflammatory and neurogenic changes on a transcriptional level, 1 and 4 h after acute ECS, and reported differences in a wide array of genes. An hour after 2 days comprising four ECS courses, the transcription of the cyclooxygenase-2 (COX2) gene, involved in acute inflammatory response, was increased in the hippocampus and hypothalamus of rats. Transcripts of cytochrome P450 (CYP450) in the hypothalamus, of CXC motif chemokine ligand 12 (CXCL12) in the hippocampus, and of IL-16 in the locus coeruleus, were decreased (Conti et al., 2007). On a neurogenic level, BDNF and Homer1 transcripts were upregulated in the prefrontal cortex as well as in the hippocampus and amygdala (Conti et al., 2007). In the second study (Altar et al., 2004), a single acute ECS treatment caused an increase in major histocompatibility complex (MHC) class 1b and CX3C motif chemokine gene expression, as well as neurogenesis-related molecules like Narp, cAMP-regulated phosphoprotein, activity regulated cytoskeleton associated protein (Arc), TrkB, early growth response 1 (EGR1) and vascular endothelial growth factor (VEGF) in healthy rat hippocampus 4 h after treatment. In the frontal cortex, acute ECS was associated with elevated gene expression of Jun proto-oncogene (Jun), CYP450 and IL-6 receptor but not with changes in genes involved in neurogenesis (Altar et al., 2004), which is unsurprising considering that the frontal cortex is not a neurogenic zone.

Taken together, these results show that the regulation of neurogenesis markers is relatively consistent, with evidence of activation/upregulation of this process, while the changes in inflammation markers seem to be less coherent, possibly because these affect a wide spread of brain areas, beyond the neurogenic niches. Nevertheless, the hippocampus and frontal cortex, critically involved in depression, have quite consistent evidence of increased inflammation following ECS. In addition to this, some of these molecules are known to exert both detrimental and beneficial effects, such as COX2, which is typically considered as a driver of inflammation in the context of depression (Chen et al., 2017), but has also been reported to stimulate VEGF production (Eibl et al., 2003) and angiogenesis in granuloma tissue (Majima et al., 2000). Similarly, CYP450 can metabolise either omega-6 or omega-3 polyunsaturated fatty acids into, respectively, pro-inflammatory or anti-inflammatory and neurogenesis-enhancing metabolites (Calderon and Kim, 2004; Giacobbe et al., 2020; Schunck et al., 2018). Therefore, this could represent a potential mechanism through which inflammation could induce neurogenic effects.

Even more interesting is the fact that despite these disparate effects, ECS would seem to upregulate inflammatory factors up until 4 h and start reducing it 6 h after acute administration. Indeed, in the last study using acute ECS and evaluating changes in inflammatory factors, the expression of calbindin 1 (Calb1), a marker of mature granule cells, and tryptophan 2,3-dioxygenase (Tdo2), which can be considered as both a marker of mature granule cells and inflammation, was found to be decreased in the hippocampus 6 h after ECS, but returned to levels comparable to control after 24 h (Imoto et al., 2017). Tdo2 is an important enzyme of the kynurenine pathway, and overactivation of this pathway has been associated with increased inflammation in depression (Sforzini et al., 2019); indeed pro-inflammatory cytokines, such as IL-1β, have been shown to upregulate Tdo2 (Borsini et al., 2017). Thus, this decrease in Tdo2 might indicate that ECS-induced inflammation would start declining at 6 h, while the number of Calb1+ mature granule cells may not increase, perhaps suggesting a shifting from cell maturation towards cell proliferation caused by ECS, as previously detected (Nakamura et al., 2013).

#### Chronic ECS treatment

Chronic ECS induced more homogenous short-term inflammatory changes than acute ECS. In the first study (Altar et al., 2004), genes associated with the innate immune system and neurogenesis were affected in rat hippocampus 4 h following the last daily ECS treatment of a 10 day course. Upregulated innate immune system genes included COX2 and prostaglandin D synthase (PGDS), while interferon-related developmental regulator 1, the role of which remains unexplored in the brain, was downregulated (Altar et al., 2004). Even though PGDS has been proposed to mediate neuroprotective mechanisms in the context of ischemic stroke (Zhang et al., 2017), it is important to notice that the production of both this enzyme and COX2 are triggered in response to acute inflammation (Ishii, 2015). For instance, COX2 is produced in response to pro-inflammatory cytokines and higher levels are associated with inflammatory processes (Myint et al., 2007), which may be induced by chronic ECS in this case.

In the same study, gene expression of BDNF, transforming growth factor beta (TGFβ), and HES-1 factor were significantly increased, when compared with the control group that did not receive ECS (Altar et al., 2004). Previous studies have shown neurogenesis to be enhanced by TGFβ (Mathieu et al., 2010) and by TGFβ+ activated microglia in the context of neurodegeneration (De Lucia et al., 2016). In addition, TGFβ has been identified as a factor inducing Notch-independent HES-1 signalling (Chacón and Rodríguez-Tébar, 2012), which would specifically contribute to the maintenance of a primitive neural stem cell pool (Dhanesh et al., 2017). This would support the idea that, as for acute ECS, chronic treatment would tend to increase the activity of the innate immune system and simultaneously contribute to proliferative neurogenic processes 4 h later.

Similar to acute ECS, chronic treatment seemed to be associated with a decline in inflammation 6 h after the last shock while still promoting proliferation of immature cells. In this next study (Imoto et al., 2017), which we previously mentioned, IL-1 receptor gene expression and cytokine response genes cluster were downregulated in the dentate gyrus together with mature granule cell markers Calb1 and Tdo2 at 6 and 24 h after ECS was applied four times per week for up to 3 weeks. These changes were maintained for the next 14 days. However, this treatment also increased the immature granule cell marker calretinin and shifted the electrophysiological profile of those cells towards a juvenile-like phenotype 24 h after the last treatment. More specifically, the somatic excitability was higher and a lower resting potential was recorded (Imoto et al., 2017). Thus, immune activation may start decreasing at this time point, as indicated by lower IL-1 receptor gene expression, cytokine response genes, as well as Tdo2. While there was a negative effect of ECS on mature neurons in this study, this is in line with the evidence examined thus far and reporting no changes in cell maturation. However, clear improvements in terms of immature and proliferating cells have been described and may reveal an ECS-induced specific shift of neurogenesis towards proliferation, therefore increasing the pool of neuronal progenitors necessary for neurogenic processes to occur.

The last study investigating short-term consequences of chronic ECS further indicates that inflammation would be dampened and controlled after the first hours following treatment. Narp knockout mice were subjected to ECS once a day for 5 days (Chang et al., 2018). Although Narp is commonly considered in the context of synapse modulation, it may also play a role in distinct immune mechanisms. It is part of the pentraxin family, as are inflammatory components such as C-reactive protein (CRP) or PTX3, which are modulated by inflammatory cytokines, like IL-1β or TNF-α (Bottazzi et al., 2016). Recently, Narp knockout mice were shown to have more Iba-1-positive activated microglia in a sciatic nerve transection model, confirming an involvement of Narp in regulating immune responses (Miskimon et al., 2014). Although this evidence is indirect, it may explain why, in this study (Chang et al., 2018), ECS reduced depression-like symptoms assessed with the tail suspension test in wild type but not knockouts, which was confirmed in animals undergoing the chronic stress paradigm. The knockouts also did not respond to ECS with increased doublecortin positive (DCX+) arborisations compared with the control group 24 h after the last shock (Chang et al., 2018). While ECS increased hippocampal BDNF mRNA and proliferating cells similarly in both groups, the behavioural differences might be attributable to the role of Narp in neurogenesis but also in the immune system. The lack of ECS response in knockouts may therefore highlight the necessity of a controlled, rather than increased, inflammatory response at 24 h post-treatment to obtain full effects on neurogenesis.

### Innate immune system and neurogenesis outcomes assessed days and weeks after acute or chronic ECS

In this section, the effects of acute or chronic ECS on innate immune system and neurogenesis outcomes will be discussed in the days and weeks following the end of treatment. Four different studies out of the 15 collected pre-clinical studies gave insight into glial activation along with neurogenesis, only after chronic ECS treatment (Table 1). Unfortunately, this time we were not able to identify studies investigating changes in inflammatory molecules as previously reported a few hours after acute or chronic ECS. Therefore, we decided to include three studies which instead investigated changes in glial proliferation, including markers of microglia, as evidence of a modulated innate immune system response, along with neurogenesis both during acute and chronic ECS (Table 3).

**Table 3.** Glial proliferation and neurogenesis after acute and chronic ECS.

Article	Animals	Experimental manipulations	ECS frequency	Sacrifice/samples	Innate immune system finding	Neurogenesis finding	Behavioural finding
**Glial proliferation and neurogenesis**							
Wennström (2003)	Wistar rats	ECS	1x	BrdU on days 0, 2, 4, 8 after ECS Sacrificed 4 weeks after ECS	↗NG2+ cells (glia marker) (peaks 2 days after ECS and cells are maintained at sacrifice) ↗Ox-42 (microglia) in molecular layer of HIPP (2d after acute ECS)	↗ NeuN/BrdU+ cells in GC layer	–
			1/day for 5 days	BrdU on days 2-6 Sacrificed on day 7	↗NG2+ cells (glia marker) (peaks 2 days after ECS and cells are maintained at sacrifice)		
Wennström (2004)	Wistar rats	ECS	1/day for 4 days	BrdU on days 2-6 Sacrificed on day 7 or 34	↗NG2+ cells (glia marker) (day 7 sacrifice, ↘observed on day 34 sacrifice) ↗Ox-42 (microglia) in medial amygdala nucleus (on day 7 and 34 sacrifices)	No change in NeuN/BrdU+ cells in amygdala (any time)	–
Öngür (2007)	Sprague-Dawley rats	ECS	1/day for 10 days	BrdU 30 min and 12h after ECS Sacrifice 4 weeks later	↗ glia (BrdU/PLP+ cells, BrdU/NG2+ cells)	↗BrdU+ cells in PFC ↘SPRY2 No BrdU/NeuN+ cells	–

↗ increase; ↘ decrease; - not assessed.

BrdU: bromodeoxyuridine; ECS: electroconvulsive shock; GC: granule cell; HIPP: hippocampus; PFC: prefrontal cortex; PLP: proteolipid protein; SPRY2: sprouty 2.

### Astroglial activation and neurogenesis

#### Chronic ECS treatment

In all four studies, astroglial activation was maintained after chronic ECS in the hippocampus from 3 days up until 5 weeks. In the first study (Dwork et al., 2004), GFAP expression was higher in the frontal gyrus, hippocampus, and amygdala of rhesus macaques 3 days after the last shock of ECS administered four times a week for 6 weeks. With regards to neurogenesis, no differences were detected in any region with regards to mature neurons labelled with microtubule-associated protein 2 (MAP2) (Dwork et al., 2004). However, another study found that GFAP protein and mRNA levels remained increased, together with BDNF protein expression, in the hippocampus of rats injected with corticosterone to model depression-like behaviour, 11 days after a daily 10 day ECS course. The efficacy of ECS was also confirmed on a behavioural level, with reduced immobility in the forced swimming task (O’Donovan et al., 2014).

Similarly, in a study using mice with an entorhinal cortex lesion used to model synaptic reorganisation (Steward, 1994), GFAP mRNA expression was increased by single daily ECS in the dentate gyrus, from 2 days after the last treatment up until 14 days, including a peak at 4 days. Cholinergic sprouting in the area of the dentate gyrus receiving entorhinal projections was, however, reduced as late as 3 weeks after (Steward, 1994). However, it is important to notice that the lesion in this model is an aggressive method to generate synaptic reorganisation and could, unsurprisingly, cause additional profound changes interfering with the effects of ECS. This is reinforced by the last study (Yao et al., 2015), in which healthy rats displayed increased GFAP after a 6 day-long single daily ECS treatment, but also Neuro-D and DCX expression in the dentate gyrus when sacrificed 5 weeks afterwards. On a behavioural level, these animals had a transient short-term memory impairment, and improved long-term memory, as measured with the Morris Water Maze task (Yao et al., 2015), which reflects the experience of patients after ECS.

Overall, these studies present a sustained astrocytic activation in response to chronic ECS, which would last not only for days but for weeks afterwards. As discussed previously, astrocyte atrophy and dysfunction are characteristic of depression, and maintaining higher levels of astrocyte activation might be a beneficial mechanism of ECS to improve depression. In consequence to this, neurogenesis would initially be enhanced by trophic factors, such as BDNF, and only come to the development of mature cells as late as 5 weeks after treatment. Even though ECS is known to have rapid behavioural effects, neurogenic processes may take some time to fully reach the maturation stage, which is not unlike the delayed action of SSRIs.

### Glial proliferation and neurogenesis

#### Acute ECS treatment

Only one study investigated the long-term effects of acute ECS on glial proliferation and the evidence indicates that the positive effects may last for several weeks (Wennström et al., 2003). In this study using single ECS in rats, treatment increased the number of proliferating cells positive with the respective glia and microglia markers NG2 and Ox42 with a peak 2 days after treatment. Three weeks later, NG2+ cells were still present in the molecular layer, granule cell layer, and hilus region of the middorsal hippocampus, which is known to be involved in the cognitive aspects of depression (Anacker and Hen, 2017). In contrast, the number of proliferating Ox42+ cells did not differ from control anymore. Nonetheless, a large proportion of the proliferating cells in the granule cell layer were positive with the neuronal marker NeuN and were more numerous than in the group which did not receive ECS (Wennström et al., 2003). While it is difficult to draw firm conclusions without additional data, acute ECS would promote lasting proliferation of NG2+ glial cells and cause changes in neuronal maturation only weeks after treatment. However, no effect of acute ECS was found for proliferating microglia.

#### Chronic ECS treatment

This enhanced glial proliferation was also present in the two studies using chronic ECS. In the first one (Wennström et al., 2004), increased proliferation of NG2+ cells was reported in several nuclei of the amygdala, and proliferation of Ox42+ cells was increased in the medial nucleus of the amygdala of healthy rats 3 days after a 5 day-long ECS course of single daily shocks. With respect to neurogenesis, no changes in the number of DCX+ cells were detected in response to ECS (Wennström et al., 2004). However, 3 weeks later the number of NG2+ cells started to return to control levels in rat amygdala after 4 days of single ECS, but the number of Ox42+ cells in the medial nucleus remained elevated even at this timepoint. Very few proliferating cells were positive to the neuronal NeuN marker (Wennström et al., 2004), which was also observed in the prefrontal cortex in the second study, subjecting animals to daily ECS for 10 days (Öngür et al., 2007). However, they reported a decreased expression of the sprouty 2 (SPY2) protein, an inhibitor of cell proliferation, in the same brain region. The presence of proliferating NG2+ and of protein proteolipid protein (PLP)+ cells, indicating oligodendrocytes precursors, was also higher 4 weeks after ECS (Öngür et al., 2007).

Although there is little evidence regarding microglia, both acute and chronic ECS treatment seem to have positive effects on glial and neuronal proliferation as well as hippocampal cell maturation in the long term, which reinforces findings indicating a beneficial role of ECS-induced gliogenesis. Although adult NG2+ glia do not retain the plastic potential that postnatal NG2+ glia possess to differentiate into oligodendrocytes, astrocytes, or neurons, previous research has pointed out that they remain in a stem cell-like state for a longer period of time and are necessary for neuronal plasticity (Viganò and Dimou, 2016). Furthermore, although the lasting effects of ECS on maturation of neurons appear heterogeneous, mature cells were observed in the hippocampus, a region most critical for neurogenesis, as opposed to the amygdala and prefrontal cortex. ECS would indeed generally affect proliferation more strongly, as denoted by its modulating actions on molecular proliferation factors and glial markers. This direction should be further investigated, particularly markers of microglia, which may also be affected by ECS and, in consequence, contribute to the regulation of inflammatory processes together with astrocytes. This may, partly, explain why ECS is so effective in patients, not only in the short term but also in the long term when, in combination with pharmacotherapy, it reduces relapse rates (Elias et al., 2017; Jelovac et al., 2013).

## Conclusion

**Figure 1.**
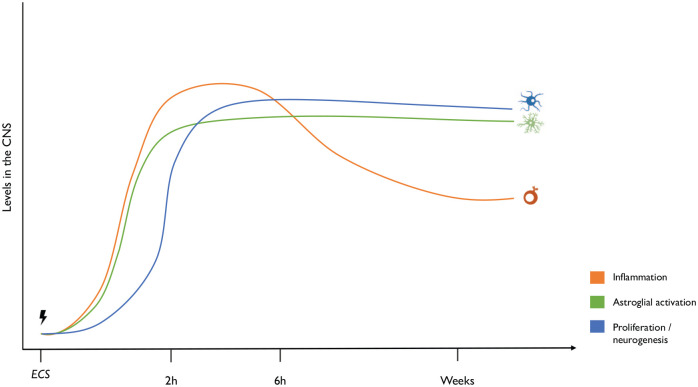
Proposed mechanism of ECS on neurogenesis, astroglial activation, and neuroinflammation over time. ECS initially increases neuroinflammation and astroglial activation, while neurogenesis, particularly proliferation, is stimulated. Inflammation starts decreasing 6 h after ECS while the effects on glia and neurogenesis remain stable.

Overall, the examined pre-clinical studies show a modulatory effect of ECS on CNS measures of the innate immune system and of neurogenesis. In the few hours following treatment, ECS leads to astrocytic activation and increase of inflammatory molecules, which is associated with an upsurge in growth and trophic factors promoting neurogenic processes. Over time, the central inflammatory response is dampened, while astrocytes remain activated and the proliferation of new cells, including neuronal and glial cells, continues, especially in the hippocampus. This mirrors the clinical finding that ECT leads to an increase in cytokines directly after treatment but tends to slowly lower baseline inflammation over the course of the sessions (Järventausta et al., 2017). An increase in innate immune activation may be involved in the regulation of neurogenesis. This may be, in part, related to the pro-neurogenic effects of immune molecules with important homoeostatic functions, including cytokines which can exert both pro- and anti-inflammatory as well as neurogenic properties (Borsini et al., 2015; Johansson et al., 2008). In addition, the lasting effect on glia, and specifically astrocytes, is particularly interesting in the aforementioned context of astrocytic atrophy in depression (Figure 1). This needs further attention, and future studies should use markers more specific than GFAP to discern whether these activated astrocytes are, for instance, neurotoxic or neuroprotective at different timepoints after ECS.

This review is not without its limitations, and it should be noted that most studies do not model depression or dysregulated inflammatory processes. This should be addressed in future research even though there are considerable challenges to ECS research, such as assessing behaviour. Nonetheless, in collecting all pre-clinical evidence reporting both immune and neurogenic outcomes in the context of ECS, this review proposes a hypothesis in which ECS enhances and maintains neurogenesis, particularly proliferation, and causes transient acute innate immune system reaction and an activation of astrocytes which could reverse astrocytic dysfunction in depression. Undeniably, the existence of human adult neurogenesis still causes intense debates, but many of the reported studies indicate increases in neurogenic molecules such as BDNF, which is considered an important player in antidepressant response. For this reason, higher levels of neurogenesis-related molecules may nevertheless be beneficial for patients and represent a mechanism whereby ECT exerts its antidepressant effects.
